# Psychometric properties of an adapted working alliance scale among Latinos

**DOI:** 10.3389/fpsyg.2025.1646855

**Published:** 2026-01-16

**Authors:** Francisco Cartujano-Barrera, Alessandra Fabrino Bretas Cupertino, Daimarelys Lara, Laisa Marcorela Andreoli Sartes, Jeffrey W. Ramos-Santiago, Ciara A. Torres, Dora Ponce, Delwyn Catley, Lisa Sanderson Cox, Ana Paula Cupertino

**Affiliations:** 1 Department of Public Health Sciences, University of Rochester Medical Center, Rochester, NY, United States; 2 Department of Nutrition, University of Brasilia, Brasilia, Brazil; 3 Department of Psychology, Federal University of Juiz de Fora, Juiz de Fora, Brazil; 4 Department of Surgery, University of Rochester Medical Center, Rochester, NY, United States; 5 The Decídetexto Community Advisory Board, Hackensack, NJ, United States; 6 Center for Children's Healthy Lifestyles & Nutrition, Children's Mercy Kansas City, Kansas City, KS, United States; 7 Department of Population Health, University of Kansas School of Medicine, Kansas City, KS, United States

**Keywords:** Latino adults, Latinos, smoking, smoking cessation, working alliance scale

## Abstract

**Objective:**

To assess the psychometric properties (i.e., internal consistency reliability, factorial validity, concurrent validity, and discriminant validity) of the Working Alliance with Smoking Cessation Program (WASCOP) Scale, an adapted scale of working alliance designed for “non-traditional” therapeutic settings.

**Methods:**

This was a secondary data analysis of the Month 3 follow-up data from *Decídetexto*, a randomized clinical trial assessing the efficacy of a culturally accommodated mobile smoking cessation intervention among Latinos. The sample included 374 participants who completed at least 10 of the 12 WASCOP items. Analyses included Cronbach’s alpha, exploratory factor analysis, and Spearman’s correlations with study satisfaction and smoking self-efficacy.

**Results:**

Following the removal of two negatively worded items, the 10-item WASCOP Scale demonstrated excellent internal consistency (*α* = 0.91). Factor analysis supported a two-factor structure. WASCOP scores were moderately correlated with program satisfaction (r = 0.530) and weakly with self-efficacy (r = 0.318), supporting construct and discriminant validity.

**Conclusion:**

The 10-item WASCOP Scale possesses acceptable internal consistency reliability, construct validity, and concurrent validity among Latinos. These findings support it potential applications in both research and clinical settings.

## Introduction

*Working alliance* is commonly defined as the collaborative and affective bond between a therapist and a client (Bordin, 1979). As an important component in facilitating behavior change, working alliance is explicitly incorporated into several contemporary behavioral treatment frameworks, including Cognitive Behavioral Therapy (Beck, 2011), Dialectical Behavior Therapy (Lynch et al., 2006), and Acceptance and Commitment Therapy (Villatte et al., 2016). Within the smoking cessation literature, working alliance has garnered considerable attention. Although methodology varies across studies, such as differences in measurement instruments, timing of assessment (e.g., following the initial session versus across multiple sessions), and abstinence endpoints, findings consistently underscore working alliance’s central role in enhancing treatment adherence and smoking outcomes (Boardman et al., 2006; Goldberg et al., 2013; He et al., 2022; Klemperer et al., 2017).

Mobile interventions are frequently employed to deliver a “counseling like” experience and have demonstrated effectiveness in supporting smoking cessation (Whittaker et al., 2019). Notably, participants often engage with these interventions in a manner that resembles interactions with a live counselor (Cartujano-Barrera et al., 2019, 2023; Orfin et al., 2025). This behavior suggests the potential formation of a working alliance with mobile interventions. Accordingly, there is a pressing need for measures that can validly assess working alliance beyond traditional, face-to-face therapeutic settings. In partnership with a Community Advisory Board, we adapted the Working Alliance Inventory – Short Version to enhance its contextual relevance for “non-traditional” therapeutic settings (Hatcher and Gillaspy, 2006). This adaptation resulted in the development of the Working Alliance with Smoking Cessation Program (WASCOP) Scale. The purpose of this study was to assess the psychometric properties (i.e., internal consistency reliability, factorial validity, concurrent validity, and discriminant validity) of the WASCOP Scale among Latinos.

## Methods

### Design

The evaluation of the psychometric properties of the WASCOP Scale was based on a secondary data analysis of the Month 3 follow-up data from the *Decídetexto* study. *Decídetexto* is a randomized clinical trial that assessed the efficacy of a culturally accommodated mobile smoking cessation intervention among Latinos. Details of *Decídetexto* have been described elsewhere (Cartujano-Barrera et al., 2020, 2025). A Community Advisory Board composed of members from the Latino community provided guidance throughout the study’s design, implementation, and evaluation, ensuring the research remained aligned with community perspectives and priorities. Study procedures were approved and monitored by the Hackensack University Medical Center (#Pro2017-0528), the University of Kansas Medical Center (IRB #STUDY00004475), and the University of Rochester Medical Center (IRB #STUDY00005080) Institutional Review Boards. ClinicalTrials.gov identifier: NCT03586596.

### Participants

Participants were recruited by *Promotores de Salud* (Community Health Workers) through community-based outreach and clinical settings. Detailed recruitment procedures have been described elsewhere (Arana-Chicas et al., 2021, 2022). Eligible individuals self-identified as Hispanic or Latino, knew how to read and speak English or Spanish, were at least 21 years of age, smoked cigarettes 3 or more days per week for at least 6 months, reported interest in quitting smoking in the next 30 days, had an active cellphone with unlimited text messaging capability, knew how to send and read text messages, and were willing to complete two study visits and assessments. Exclusion criteria included use of other tobacco products more than once per week; current participation in any other smoking cessation program or use of any medication to quit smoking; living with a current study participant; being pregnant, breastfeeding, or planning to become pregnant in the next year; or planning to move in the next 6 months. For this study, participants who completed at least 10 of the 12 WASCOP Scale items were included in the analysis, resulting in a sample of 374 participants.

### Measures

Participants completed an in-person baseline survey and an over-the-phone follow-up survey at Month 3. All measures were available in English and Spanish and were administered by trained members of the research team. The baseline survey collected data on participants’ age, gender, educational attainment, preferred language, country or region of birth, and average number of cigarettes smoked per day. The Month 3 follow-up survey, administered to all participants regardless of randomization group, included the WASCOP Scale, the Smoking Self-Efficacy Questionnaire (SEQ-12), and a single-item measure of satisfaction with the smoking cessation program. The SEQ-12 is a validated 12-item instrument designed to assess an individual’s confidence in their ability to refrain from smoking across a range of different situations (Cartujano-Barrera et al., 2021; Etter et al., 2000). Each item is rated on a 5-point Likert scale, ranging from “Not at all sure” to “Absolutely sure.” Program satisfaction was assessed with the question “How satisfied are you with this smoking cessation program?” Responses ranged on a five-point scale from “extremely unsatisfied” to “extremely satisfied”.

### The working alliance with smoking cessation program (WASCOP) scale

The Working Alliance Inventory – Short Version (WAI-S) served as the foundation for the new WASCOP Scale. The WAI-S compromises 12 items, each rated on a 7-point Likert scale ranging from 1 (never) to 7 (always) (Hatcher and Gillaspy, 2006). The WAI-S is organized into three subscales: (a) *Agreement on Goals*, assessing the degree of concordance between the patient and therapist regarding the overarching treatment goals; (b) *Agreement on Tasks*, evaluating the extent to which the patient and therapist agree upon the tasks deemed necessary to achieve these goals and; (c) *Bond Between Therapist and Client*, measuring the emotional bond between the patient and therapist, encompassing elements of trust, acceptance, and attachment (Hatcher and Gillaspy, 2006).

In partnership with the Community Advisory Board, the WAI-S was adapted to reflect the structure of the *Decídetexto* study, in which participants did not interact with a traditional, face-to-face therapist. Instead, participants either received a mobile intervention or a printed educational material, which included information on accessing free behavioral counseling through their state quitline (Cartujano-Barrera et al., 2020, 2025). To ensure contextual relevance, the term “therapist” was replaced with “my smoking cessation program” throughout the scale.

A consensus approach was employed to translate the scale into Spanish. Two bilingual (English–Spanish) members of the research team independently translated the adapted English version of the scale. A third bilingual team member reviewed both translations, discussed any discrepancies with the translators, and facilitated the resolution of differences to reach consensus on the final Spanish version.

### Analysis

Simple frequencies were calculated for categorical variables and means and standard deviations for continuous variables. Internal consistency reliability of the WASCOP Scale was assessed using Cronbach’s alpha, with values greater than 0.90 indicating excellent reliability (Schmitt, 1996; Streiner, 2003). Item-total correlations were also assessed, with values below 0.30 indicating inadequate correlation with the overall scale (Ferketich, 1991; Henrysson, 1963). For factorial validity, the Kaiser-Meyer-Olkin (KMO) measure of sampling adequacy and Bartlett’s Test of Sphericity were conducted to evaluate the suitability of the data for factor analysis. A KMO value greater than 0.60 was considered acceptable (Kaiser, 1974; Bartlett, 1937). Given the developmental nature of the WASCOP Scale, an exploratory factor analysis (EFA) was conducted to examine its underlying structure. Principal component extraction with varimax rotation was used to enhance interpretability. Factors were retained based on the Kaiser criterion, which recommends retaining components with eigenvalues greater than 1.0 (Kaiser, 1960). Factor loadings of 0.40 or higher were considered indicative of meaningful item-factor associations (Stevens, 2002). For convergent validity, Spearman’s correlation was conducted to assess the correlation between WASCOP Scale scores and study satisfaction. For discriminant validity, Spearman’s correlation was conducted to assess the correlation between WASCOP Scale scores and SEQ-12 scores. Missing data were handled using complete-case analysis. No imputation was performed. Analyses were performed using the Statistical Package for the Social Sciences v29.0 (SPSS; IBM Corp, 2022).

## Results

The 374 participants included in the current analysis had an average age of 48.8 years old (SD 11.1), 45.7% were female, and 72.2% had completed at least high school education or more (Table 1). In terms of country/region of birth, 30.7% were from the Caribbean, 20.9% from Central America and Mexico, 23.2% from South America, and 25.1% were born in the United States. Additionally, over half of the participants (53.7%) smoked 10 or more cigarettes per day. Table 2 presents the content of the WASCOP Scale along with its Spanish translation.

**Table 1:** Baseline characteristics of participants (*N* = 374).

Characteristic	*n* (%)
Age, mean (SD)	48.8 (11.1)
Female	171 (45.7%)
Education ≥ high school	270 (72.2%)
Spanish as primary language	261 (69.8%)
Country/region of birth	
Caribbean^a^	115 (30.7%)
Central America and Mexico^b^	78 (20.9%)
South America^c^	87 (23.2%)
United States	94 (25.1%)
Heavy smoking^d^	201 (53.7%)

SD = Standard deviation.

a Participants who were born in Cuba, Dominican Republic, and Puerto Rico were categorized as “Caribbean”.

b Participants who were born in Belize, Costa Rica, El Salvador, Guatemala, Honduras, Nicaragua, and Panama were categorized as “Central America”.

c Participants who were born in Argentina, Bolivia, Brazil, Chile, Colombia, Ecuador, Guyana, Paraguay, Peru, Uruguay, and Venezuela were categorized as “South America”.

d Heavy smoking was defined as smoking ≥ 10 cigarettes per day.

**Table 2:** The working alliance with smoking cessation program (WASCOP) scale.

Item	Content
1	The smoking cessation program and I agree about the things I will need to do to help improve my situation. El programa para dejar de fumar y yo estamos de acuerdo sobre lo que tengo que hacer para mejorar mi situación.
2	What the smoking cessation program and I address gives me new ways of looking at my problem. Lo que el programa para dejar de fumar y yo hacemos me proporciona nuevos puntos de vista sobre mi problema.
3	I feel liked in my smoking cessation program. Creo que les caigo bien en el programa para dejar de fumar.
4	My smoking cessation program does not take into account what I am trying to accomplish. Mi programa para dejar de fumar no entiende lo que estoy tratando de conseguir.
5	I am confident in my smoking cessation program’s ability to help me. Confío en la capacidad del programa para dejar de fumar para ayudarme.
6	My smoking cessation program and I are working towards mutually agreed upon goals. Mi programa para dejar de fumar y yo estamos trabajando para conseguir los objetivos que hemos acordado.
7	I feel appreciated in my smoking cessation program. Creo que mi programa para dejar de fumar me aprecia.
8	My smoking cessation program and I agree on what is important for me to work on. Mi programa para dejar de fumar y yo estamos de acuerdo sobre lo que es importante que yo trabaje.
9	I trust my smoking cessation program. Yo confió en mi programa para dejar de fumar.
10	My smoking cessation program and I have different ideas on what my problems are. Mi programa para dejar de fumar y yo tenemos distintas ideas sobre cuáles son mis verdaderos problemas.
11	My smoking cessation program and I understand the kind of changes that would be good for me. Mi programa para dejar de fumar y yo entendemos qué tipo de cambios me vendrían bien.
12	I believe the way we are working with my problem is correct. Creo que estamos trabajando en mi problema de forma adecuada.

Responses ranged on the following seven-point scale: 1 = Never, Nunca; 2 = Rarely, Raramente; 3 = Occasionally, Ocasionalmente; 4 = Sometimes, A veces; 5 = Often, Seguido; 6 = Very often, Muy seguido; 7 = Always, Siempre. Items 4 and 10 were removed due to their low item-total correlations and improvements in internal consistency reliability upon their removal.

### Internal consistency reliability

The Cronbach’s alpha coefficient for the total scale was 0.81. The item-total correlation matrix is detailed in Table 3. All items had item-total correlation values above 0.55, except for item 4 (0.159) and Item 10 (0.031). The overall Cronbach’s alpha decreased when Items 1, 2, 3, 5, 6, 7, 8, 9, or 11 were removed. In contrast, removing Item 4 or Item 10 increased the alpha to 0.85 and 0.86, respectively.

**Table 3:** Item-total correlations and Cronbach’s alpha if item deleted for each item of the 12-item WASCOP scale.

Item	Item-total correlation	Cronbach’s Alpha if item deleted
1	0.555	0.799
2	0.592	0.796
3	0.578	0.798
4	0.159	0.853
5	0.659	0.793
6	0.696	0.791
7	0.664	0.795
8	0.669	0.794
9	0.759	0.788
10	0.031	0.866
11	0.596	0.797
12	0.695	0.792

Internal consistency reliability was recalculated after removing items 4 and 10, resulting in an increased Cronbach’s alpha coefficient of 0.91 for the total scale. The new item-total correlation matrix is detailed in Table 4. All items had item-total correlation values above 0.55. Cronbach’s alpha coefficients did not increase with the removal of any additional items.

**Table 4:** Item-total correlations and Cronbach’s alpha if item deleted for each item of the 10-item WASCOP scale.

Item	Item-total correlation	Cronbach’s Alpha if item deleted
1	0.553	0.918
2	0.623	0.913
3	0.635	0.911
5	0.748	0.904
6	0.732	0.905
7	0.725	0.906
8	0.734	0.905
9	0.825	0.901
11	0.666	0.909
12	0.759	0.904

### Construct validity

Construct validity of the 10-item scale (following the removal of items 4 and 10) was evaluated. The Kaiser-Meyer-Olkin measure of sampling adequacy was 0.92, indicating suitability for factor analysis. The Bartlett’s Test of Sphericity was significant (x^2^ = 2191.75, d.f. = 45, *p* < 0.001), confirming that the correlation matrix was factorable. Two factors were extracted, explaining 38.31 and 19.16% of the variance, respectively. Together, the two factors accounted for 57.47% of the total variance. Items 3, 5, 6, 7, and 9 loaded on Factor 1. Items 1, 2, 8, and 12 loaded on Factor 2.

A moderate to strong positive correlation was observed between WASCOP Scale scores and study satisfaction (r = 0.530; *p* = 0.001). A weak to moderate positive correlation was observed between WASCOP Scale scores and SEQ-12 scores (r = 0.318; *p* = 0.001).

## Discussion

This study assessed the psychometric properties of the WASCOP Scale among Latinos who completed a smoking cessation randomized clinical trial. The WASCOP Scale is an adaptation of the WAI-S, designed to assess working alliance across “non-traditional” therapeutic settings, such as mobile health interventions. The 10-item scale is available in both English and Spanish and was developed in partnership with a Community Advisory Board. Findings demonstrated that the WASCOP Scale possesses acceptable internal consistency reliability, factorial validity, concurrent validity, and discriminant validity among Latinos.

Although the original WAI-S included two negatively worded items to reduce response bias and promote respondent attentiveness (Hatcher and Gillaspy, 2006), those items were removed in this study. Their removal was justified by low item-total correlations and improvements in internal consistency reliability upon their removal. Specifically, items 4 (“My smoking cessation program does not take into account what I am trying to accomplish”) and 10 (“My smoking cessation program and I have different ideas on what my problems are”) both assessed relational discord and were embedded within a predominantly positively framed item set. This contrast may have contributed to participant confusion or measurement inconsistency, thereby diminishing their psychometric utility. This finding is in line with prior research suggesting that negatively worded items adversely affect the psychometric performance of scales among Latino adults (Venta et al., 2022).

Factor 1 encompassed items from the original subscales *Agreement on Goals* and *Bond Between Therapist and Client*. This suggests a shared underlying dimension of interpersonal connection and mutual understanding within the therapeutic relationship. Factor 2 retained all items from the original *Agreement on Tasks* subscale, supporting the conceptual distinctiveness and structural integrity of this construct. These findings provide support for the notion that participants can perceive a therapeutic alliance with a “smoking cessation program” as they do with a therapist.

The moderate to strong positive correlation between WASCOP Scale scores and study satisfaction provides preliminary evidence of convergent validity. This association is consistent with existing literature demonstrating that higher levels of working alliance are associated with increased patient satisfaction across various healthcare contexts (Kim et al., 2008; MacInnes et al., 2014; Sylvia et al., 2013). These findings extend the potential critical role of working alliance to therapeutic contexts beyond face-to-face counseling. The weak to moderate positive correlation between WASCOP Scale scores and SEQ-12 scores provides preliminary evidence of discriminant validity. This finding suggests that the WASCOP Scale measures a construct that is conceptually distinct from self-efficacy.

### Strengths and limitations

This study has several notable strengths. First, the sample size was appropriate for the psychometric analyses conducted. Second, the diversity among participants supports the relevance and applicability of the scale in the Latino community. Third, the availability of the scale in Spanish improves accessibility for Spanish-speaking individuals. Lastly, a Community Advisory Board was actively engaged in the development, implementation, and analysis phases, which strengthens the cultural appropriateness of the scale.

Despite the noted strengths, this study has some limitations that must be considered. First, the present study did not assess the predictive validity of the WASCOP Scale or its potential mediating role in smoking cessation outcomes. As this analysis focused on preliminary psychometric validation, future research should explore these dimensions to further establish the scale’s utility in intervention studies. Second, the analysis was not stratified by intervention arm. Half of the participants received a mobile smoking cessation intervention, while the other half received a printed educational material and access free behavioral counseling (Cartujano-Barrera et al., 2020, 2025). Given the distinct nature of these interventions, participants likely experienced varying levels of working alliance. However, the decision not to conduct separate analyses by intervention arm is appropriate for psychometric evaluation, as it captures a broader range of working alliance levels, thereby enhancing the robustness of the scale’s validation. Lastly, given that the WASCOP Scale was administered by the research team, there is a potential for social desirability bias among participants (Fisher, 1993). However, the research team administering the WASCOP Scale was distinct from the research team managing the mobile intervention, and participants were explicitly informed that they were free to express their honest opinions.

## Conclusion

The WASCOP Scale is an adaptation of the WAI-S, developed to assess working alliance across “non-traditional” therapeutic settings. The 10-item scale possesses acceptable internal consistency reliability, construct validity, and concurrent validity among Latinos. These findings support it potential applications in both research and clinical settings to assess working alliance across “non-traditional” therapeutic settings, such as mobile health interventions.

## Data Availability

The data analyzed in this study is subject to the following licenses/restrictions: the data generated in this study are available upon request from the corresponding author. Requests to access these datasets should be directed to FC-B, francisco_cartujano@urmc.rochester.edu.
